# Structure of frequency-interacting RNA helicase from *Neurospora crassa* reveals high flexibility in a domain critical for circadian rhythm and RNA surveillance

**DOI:** 10.1371/journal.pone.0196642

**Published:** 2018-05-02

**Authors:** Yalemi Morales, Keith J. Olsen, Jacqueline M. Bulcher, Sean J. Johnson

**Affiliations:** Department of Chemistry and Biochemistry, Utah State University, Logan, Utah, United States of America; Tulane University, UNITED STATES

## Abstract

The FRH (frequency-interacting RNA helicase) protein is the *Neurospora crassa* homolog of yeast Mtr4, an essential RNA helicase that plays a central role in RNA metabolism as an activator of the nuclear RNA exosome. FRH is also a required component of the circadian clock, mediating protein interactions that result in the rhythmic repression of gene expression. Here we show that FRH unwinds RNA substrates *in vitro* with a kinetic profile similar to Mtr4, indicating that while FRH has acquired additional functionality, its core helicase function remains intact. In contrast with the earlier FRH structures, a new crystal form of FRH results in an ATP binding site that is undisturbed by crystal contacts and adopts a conformation consistent with nucleotide binding and hydrolysis. Strikingly, this new FRH structure adopts an arch domain conformation that is dramatically altered from previous structures. Comparison of the existing FRH structures reveals conserved hinge points that appear to facilitate arch motion. Regions in the arch have been previously shown to mediate a variety of protein-protein interactions critical for RNA surveillance and circadian clock functions. The conformational changes highlighted in the FRH structures provide a platform for investigating the relationship between arch dynamics and Mtr4/FRH function.

## Introduction

*Neurospora crassa* FRH (frequency-interacting RNA helicase) is a 124 kDa homolog of *Saccharomyces cerevisiae* Mtr4 (56% sequence identity, 73% similarity), a Ski2-like ATP-dependent RNA helicase that plays a central role in activating the nuclear exosome to promote processing or complete degradation of RNA substrates [1–5]. FRH has been extensively characterized as an essential component of the circadian clock in *N*. *crassa* [6–9], a function which doesn’t exist in *S*. *cerevisiae* and is not dependent on enzymatic activity [10]. This unique FRH clock function appears to be primarily one of scaffolding for the other clock components. Briefly, FRH binds and stabilizes the intrinsically disordered protein and negative clock element, FRQ, to form the FRQ-FRH complex (FFC) [6, 10]. The FFC binds to the positive transcriptional regulators that make up the White Collar complex (WCC), White Collar 1 (WC1) and White Collar 2 (WC2), which stimulate the expression of clock-controlled genes when not bound by the FFC [11, 12]. Extensive phosphorylation of both FRQ and WCC by casein kinases 1a and 2 (CK1a and CKII) leads to eventual complex dissolution [13–15]; FRQ is targeted for degradation and the WCC complex is free to promote transcription of clock genes, including *frq*, thus re-initializing the circadian cycle which lasts approximately 22 hours [9, 16, 17].

Although FRH has been described as an RNA helicase based on its similarity to Mtr4 [6, 8, 10, 18], the unwinding activity of FRH has never been characterized. Like Mtr4, mutations predicted to disrupt helicase activity do not support cell viability [10]. FRH interacts functionally with exosome components to promote degradation of RNA transcripts, including *frq* [7]. Homologs of other Mtr4 interacting proteins, such as Trf4 and Air2, have been identified in *N*. *crassa*, but direct interactions with FRH have not yet been reported [9, 19]. Significantly, while FRH is required for viability [6], mutations that disrupt the circadian clock do not affect cell survival [8], suggesting that the essential function of FRH is related to its role in RNA surveillance rather than the circadian clock. It is not known whether the additional circadian function of FRH has any impact on helicase activity.

The crystal structure of FRH was recently determined in apo and ADP bound states [19]. Like previously determined structures of Mtr4 from *Saccharomyces cerevisiae* [20–22], FRH contains a largely unstructured (169 amino acid) N-terminus and five structured domains: two RecA-like domains, a small winged helix domain, a helical “ratchet” or DSHCT domain, and an arch-like insertion domain that includes a KOW module. The first four domains form a core ring-like structure with the arch domain spanning one side of the core.

The arch domain is a defining feature of Mtr4 and FRH structures. Although sequence conservation is limited throughout the arch, the domain architecture appears to be conserved [19, 20]. The domain is composed of two anti-parallel alpha helical coiled coil segments (arm and forearm) that terminate with a β-barrel fold (fist) containing a KOW module. Comparison of the existing crystal structures reveals a range of arch conformations, suggesting that it is a flexible domain. For example, FRH was previously crystallized in an orthorhombic space group with two unique cell dimensions (“large cell” and “small cell”). The differences in crystal packing resulted in two distinct arch conformations, while the remaining core domains (RecA1, RecA2, winged helix and helical domains) were relatively undisturbed [19]. The function of the arch is not fully understood, but deletion of the arch domain results in a severe growth defect and stalled processing of the 5.8S rRNA in *S*. *cerevisiae* [20]. In Mtr4, the arch plays an important role in RNA unwinding [23] and has the ability to interact directly with RNA substrates such as tRNA [21, 24] and rRNA [20, 25]. The fist/KOW module of Mtr4 (and presumably FRH) interacts with accessory proteins containing an AIM (arch interaction motif) sequence [25]. Residues in this region of the arch are also required for FRH-WCC interaction [6, 8].

Here we present a new crystal structure of FRH. The structure reveals a conformational rearrangement of the arch beyond what has been observed in other structures, providing a more complete description of the range of motions accessible to this domain. Multiple conserved hinge points are identified that appear to facilitate domain rearrangement. In contrast with previously published FRH structures, the ATP binding site is undisturbed by crystal contacts and adopts a conformation compatible with ATP binding and hydrolysis. We also present the first kinetic characterization of FRH, directly demonstrating that FRH unwinds RNA substrates with an activity and sequence specificity similar to Mtr4.

## Results and discussion

The full length FRH protein was recombinantly expressed in *E*. *coli* with a cleavable N-terminal GST tag. Purification included GST and heparin affinity chromatography, removal of the GST tag by TEV cleavage, DEAE and size exclusion chromatography. The purified protein was crystallized in the presence of 0.2 M Sodium Citrate pH 5.6 and 19% PEG 3350, resulting in a trigonal P3_1_21 crystal form (FRH^Trig^) (Table 1) that is unique from the previously published orthorhombic P2_1_2_1_2_1_ FRH structures (FRH^Ortho^) [19]. Data were collected to 3.5 Å resolution and the structure was determined using Mtr4 as a molecular replacement search model (PDB 4U4C, sequence identity 53%). An updated sequence alignment based on the existing Mtr4 and FRH structures is provided in S1 Fig.

**Table 1:** Data collection and refinement statistics.

	FRH^Trig^ (PDB ID 6BB8)
**Data collection**	
Source	SSRL 14–1
Space group	P3_1_21
Cell dimensions	
*a*, *b*, *c* (Å)	117.77, 117.77, 180.41
α, β, γ (°)	90.0, 90.0, 120.0
Resolution (Å)	30.0–3.50 (3.65–3.50)*
*CC*_1/2_	0.564
*I* / σ*I*	28.3 (0.93)
Completeness (%)	99.9 (98.7)
Redundancy	22.1 (22.4)
**Refinement**	
No. reflections	18,697 (2355)
*R*_work_ / *R*_free_	0.251/0.301
No. atoms	
Protein	7225
*B*-factors	
Protein	173.6
R.m.s deviations	
Bond lengths (Å)	0.007
Bond angles (°)	1.428
Ramachandran	
Favored (%)	89.01
Allowed (%)	7.7
Outliers (%)	3.3

*Values in parentheses are for highest-resolution shell.

**Fig 1:**
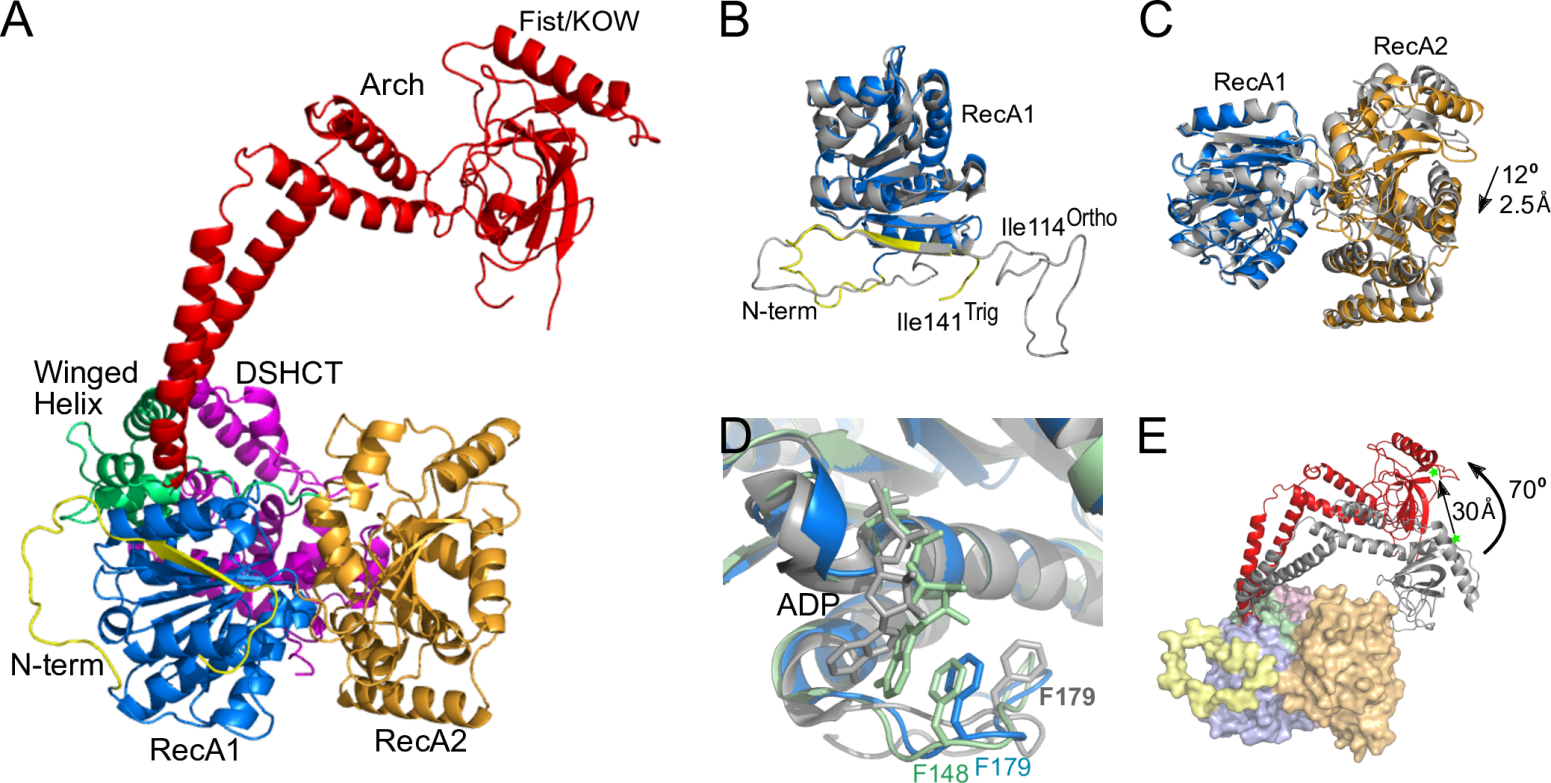
Comparison of FRH^Trig^ and FRH^Ortho^ structures. (A) The FRH^Trig^ structure (PDB ID 6BB8) colored by domain. N-terminal region (yellow), RecA1 (marine), RecA2 (orange), Winged Helix (green), Arch (red), and DSHCT (purple). (B-E) Superposition of FRH^Trig^ (colored as in (A)) and the FRH^Ortho^ small cell (gray; PDB ID 5E02 structures. (B) The observed N-terminal regions differ in both length and conformation. (C) A small shift is observed in the relative position of the RecA domains. (D) A close-up view of the nucleotide binding site in the RecA1 domain shows that the FRH^Trig^ structure (blue) more closely resembles the conformation observed in an ADP bound form of Mtr4 (light green, PDB ID 2XGT) than the more open FRH^Ortho^-ADP structure (gray, PDB ID 5E02). (E) Superposition of the FRH^Trig^ and FRH^Ortho^ small cell structures reveals significant repositioning of the arch domain, including a 70° rotation of the fist/KOW module. This results in a 30 Å displacement of R806 (green stars), a residue implicated in both White Collar Complex (WCC) and arch interaction motif (AIM) interactions. Figures were rendered using PyMOL [26].

The newly determined FRH^Trig^ structure retains the same general architecture as the previously determined FRH^Ortho^ structures. However, several notable differences are observed (Fig 1). (For clarity, we reference differences with the apo FRH^Ortho^ large cell structure (PDB ID 4XGT) unless otherwise noted.) (1) The N-terminal region adopts a random coil structure that deviates substantially from the previous structure (Fig 1B). The visible N-terminal region is shorter than that observed in the FRH^Ortho^ structures, beginning at residue 141 for FRH^Trig^ as opposed to residue 114 for FRH^Ortho^. In both cases, the conformation of the N-terminus appears to be largely influenced by crystal contacts. (2) While the overall structure of the RecA domains is the same (RMSD = 0.487 Å for RecA1; 0.618 Å for RecA2), the relative position of the RecA domains is slightly altered. In the FRH^Trig^ structure, the RecA domains open slightly (12° rotation, 2.5 Å translation) with respect to each other as compared to the FRH^Ortho^ structure (Fig 1C). Furthermore, rearrangements in the ATP binding site in RecA1 result in a conformation that is consistent with productive nucleotide binding and hydrolysis as observed in nucleotide bound Mtr4 structures (Fig 1D) [21]. This region was significantly disrupted by crystal contacts in the FRH^Ortho^ structures [19]. Thus, the FRH^Trig^ structure appears to be more representative of the native nucleotide binding conformation. (3) The most prominent difference between the two FRH^Trig^ and FRH^Otrho^ structures is the conformation of the arch domain. (Although the arch position also differs between the FRH^Ortho^ large cell and small cell structures, the position of the arch in FRH^Trig^ is fundamentally different from each of the FRH^Ortho^ structures.) Rotations about multiple hinge points in the arch result in a 30 Å displacement and a 70° rotation of the KOW module with respect to the FRH^Ortho^ small cell position (Fig 1E).

### Movement in the arch domain is facilitated by multiple conserved hinge points

**Fig 2:**
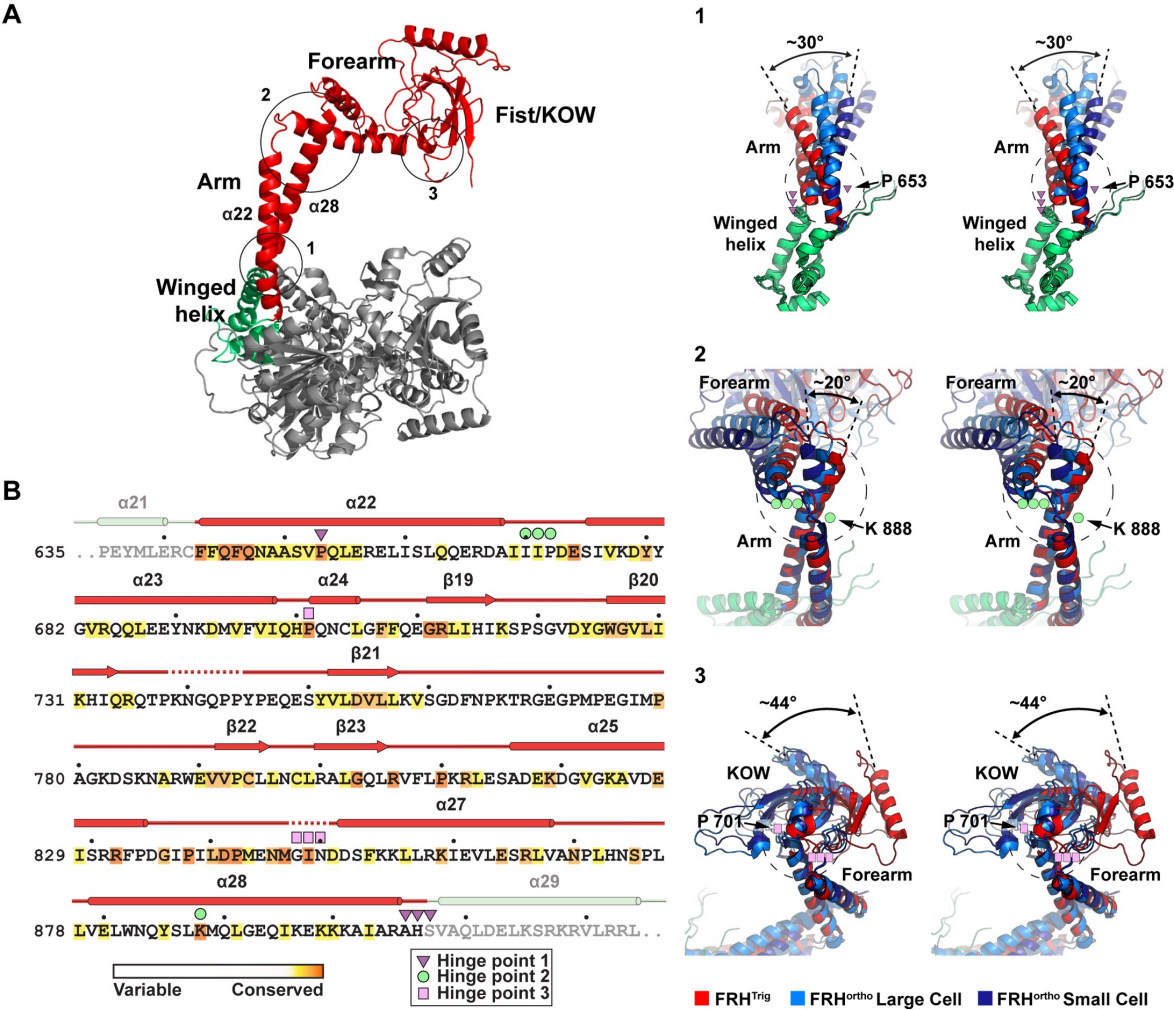
Conserved hinge points in the arch domain. (A) The FRH^Trig^ structure is shown (left) with approximate hinge points circled. Zoomed in stereo views of each hinge point are shown on the right with FRH^Ortho^ structures (large cell PDB ID 4XGT, light blue; small cell PDB ID 5E02, dark blue) superimposed on the FRH^Trig^ structure (red). In each view, the region below the hinge point was aligned to highlight the displacement above the hinge point. (B) The FRH arch domain sequence is shown colored by sequence conservation, based on an alignment of 108 FRH/Mtr4 sequences as reported in Jackson, *et*. *al*. [20] Observed secondary structure is displayed above the sequence. Identified hinge point residues are indicated by the triangle, circle and square symbols. Each hinge point contains a strictly conserved residue (dark orange) with a loop structure on the opposing strand.

Structural alignment of the FRH^Trig^ and FRH^Ortho^ structures along segments of the arch domain highlights the presence of three distinct hinge points that appear to facilitate movement in the arch (Fig 2). Hinge points are observed (1) at the base of the arch where it projects out from the winged helix domain, (2) at the junction between the arm and the forearm, and (3) at the junction between the forearm and the fist (containing the KOW module). Each hinge point contains a strictly conserved residue on one side and a loop on the opposite side (Fig 2B). While the loops generally exhibit minimal sequence conservation, the loop structures themselves are conserved between *N*. *crassa* and *S*. *cerevisiae*, and secondary structure prediction suggests that they are conserved throughout eukaryotic species (data not shown). The presence of strictly conserved residues at each hinge point is significant given the minimal sequence conservation throughout the arch; only 7% (19 out of 265) of residues in the arch are strictly conserved (based on an alignment of 108 FRH/Mtr4 sequences [20]. Notably, hinge points 1 and 2 involve 30° and 20° bends within extended helices, respectively.

Although the precise orientation of the arch in each crystal form is clearly influenced by crystal contacts (see discussion below), these structures provide a glimpse into the dynamic range of motions that are possible. Some flexibility is similarly evident in previously published Mtr4 crystal structures [20–22]. Interestingly, the analogous region in Ski2 (the cytosolic homolog of Mtr4) is moved out of the way to permit mRNA binding to the helicase core in a ribosome-Ski2-Ski3-Ski8 complex [27]. While the precise mechanistic implications of arch motion in FRH and Mtr4 are not understood, it seems likely that arch repositioning has an important effect on helicase function. The fist/KOW module binds RNA substrates [20, 21, 24] and contains a docking site for various accessory proteins containing an AIM (arch interaction motif) sequence such as Nop53 and Utp18 [25]. The fist/KOW module also appears to be functional in the circadian clock; R806 (a strictly conserved residue in the AIM binding region of the fist/KOW) is required for FRH-WCC interaction [6, 8]. Comparison of the FRH crystal structures shows displacement of R806 by up to 30 Å (Fig 1E). Notably, the local conformation and accessibility of the AIM binding region is unchanged. Thus, in the absence of additional structural data, we do not expect that conformational changes in the arch will necessarily alter the local binding of the WCC (or other accessory proteins or RNA) to the fist/KOW. However, repositioning of binding partners through arch motion are expected to alter other long-range interactions that may significantly impact both helicase and circadian clock functions of FRH.

### FRH^Trig^ forms a crystallographic dimer

**Fig 3:**
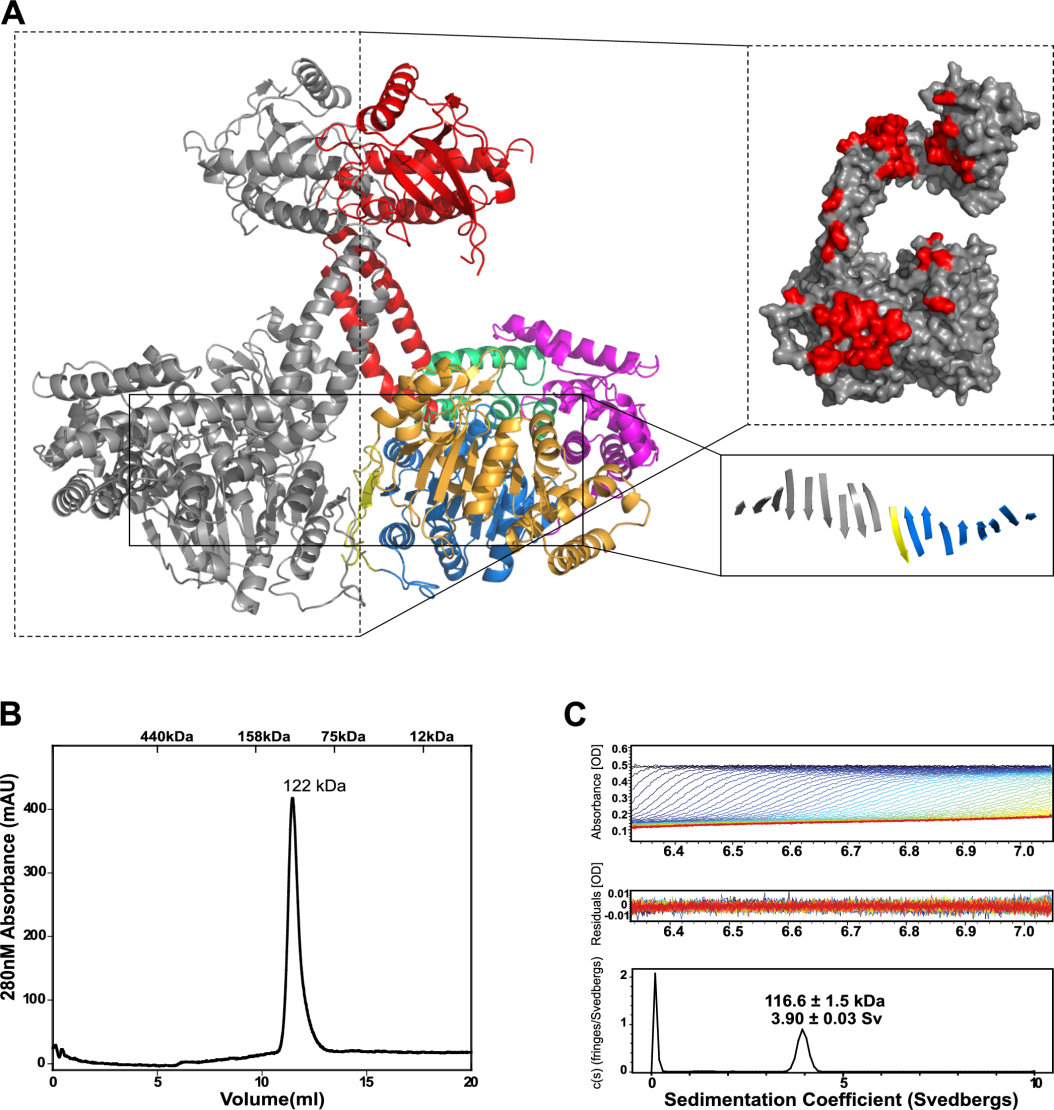
Oligomeric state analysis of FRH. (A) A crystallographic symmetry mate in the FRH^Trig^ structure (gray) forms extensive contacts with the FRH monomer, resulting in a buried surface area of 2445 Å^2^ and extension of a β sheet from the RecA1 domains and N-terminal regions of both symmetry-related molecules (bottom insert). Residues making direct contact with the adjacent FRH subunit are highlighted in red (top insert). (B) Full length FRH elutes as a single species with an estimated molecular weight of 122 kDa as determined by size exclusion chromatography, suggesting that FRH is a monomer in solution. Elution volumes corresponding to protein molecular mass standards are included at the top of the chromatogram. (C) Sedimentation velocity analytical ultracentrifugation analysis also indicates that FRH sediments as a single species, with a sedimentation coefficient of 3.90 ± 0.03 S, corresponding to a calculated molecular mass of 116.6 ± 1.5 kDa, consistent with a monomer. Representative A_280_ absorption scans, residuals from fitting the data to a continuous c(s) distribution model, and sedimentation coefficient distribution (c(s) versus S) of purified full length FRH protein are shown.

FRH^Trig^ forms extensive interactions with a crystallographic symmetry mate along a 2-fold axis, resulting in a buried surface area of ~2445 Å^2^ along the N-terminus, RecA1, RecA2 and arch domains (Fig 3). This packing creates an extended β-sheet involving symmetry related RecA1 domains. Symmetry related regions along the elbow, forearm and fist/KOW of the arch domain interlock to stabilize the extended conformation of the arch domain, including the 70° rotation of the fist/KOW (as compared to the FRH^Ortho^ structures). Notably, while stabilizing the fist/KOW rotation, this packing would not prevent the fist/KOW from adopting the conformation seen in the FRH^Ortho^ structures. The interaction surface along the arch is composed of hydrophobic and electrostatic interactions, although the residues involved in this interaction are not conserved. PISA analysis [28] of the dimer gives a complex significance score of 1.0, which suggests that the observed interaction may be biologically relevant. This observation was surprising given that all previous studies of FRH and Mtr4 suggested that these helicases exist as monomeric species [6, 20, 21], with one exception that suggested a higher order stoichiometry for the FRH-FRQ complex [18].

In order to clarify the multimeric state of FRH *in vitro*, size exclusion chromatography was performed. FRH migrates as a single species at a molecular weight of ~122 kDa, consistent with a monomer (Fig 3B). Sedimentation velocity was then monitored by analytical ultracentrifugation. FRH sedimentation results in a single peak that is also consistent with a monomeric species (Fig 3C). We note that small angle X-ray scattering (SAXS) performed by Conrad, *et al*., as well as our lab (data not shown), also supports a monomer in solution [19]. We conclude that FRH is a monomer in solution and the structural dimer observed in the FRH^Trig^ crystal is likely a crystallization artifact, although we cannot rule out the possibility that such a conformation may be biologically relevant under some conditions not yet identified.

### FRH unwinding activity

**Fig 4:**
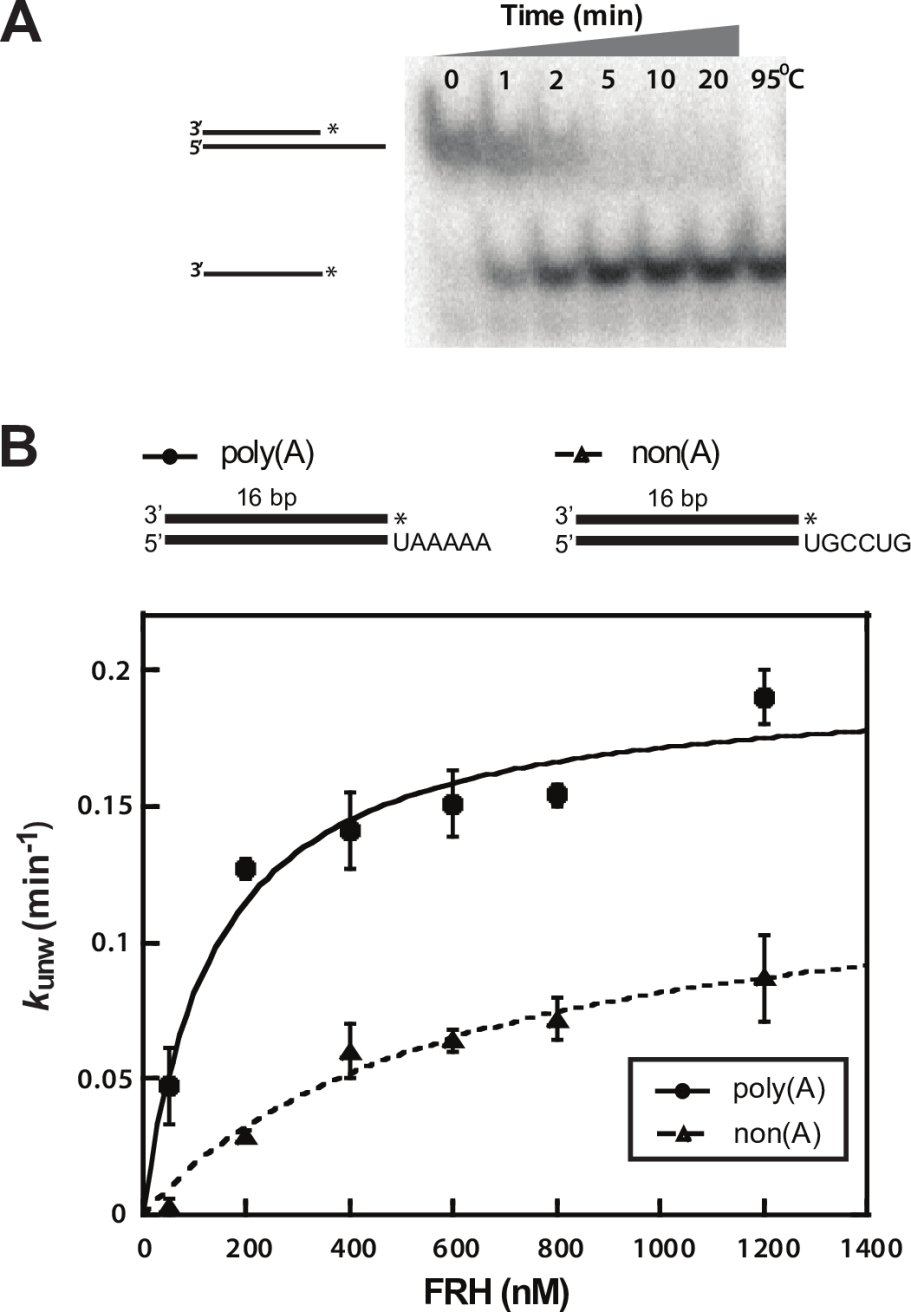
FRH unwinds RNA substrates with a preference for a poly(A) 3’ single-stranded overhang. (A) A representative unwinding assay showing displacement over time of a radiolabeled single-stranded RNA from a 16-bp duplex with a 3’ single stranded overhang by full-length FRH, as observed on a non-denaturing polyacrylamide gel. The right-most lane indicates the position of a completely denatured RNA strand, heated to 95°C. (B) Poly(A) (●) and non(A) (▲) RNA unwinding rate constants (*k*_unw_) plotted as a function of FRH concentration. Best fit curves to the data were calculated as described in ‘Materials and Methods’. Data presented corresponds to the average from three independent experiments; error bars represent SD.

Due to the sequence similarity between FRH and Mtr4, it has been assumed that FRH is a functional exosome-activating helicase, in addition to its role in the *Neurospora* circadian rhythm. However, the RNA unwinding activity of FRH has not yet been characterized. We therefore performed pre-steady state unwinding assays on full-length FRH by tracking the displacement of a ^32^P-labeled 16-nucleotide strand from a complementary strand with a 3’ single-stranded overhang of six nucleotides (Fig 4), as described previously for Mtr4 and other helicases [23, 29]. FRH, like Mtr4, was unable to unwind a substrate without a 3’ overhang (data not shown). Unwinding rate constants (*k*_unw_) were measured at several enzyme concentrations to obtain the strand-separation rate constants at enzyme saturation (*k*_unw_^max^), as well as the observed functional affinity for the substrate (*K*_1/2_). Using a 3’ polyadenylated substrate (poly(A)), FRH was able to unwind the duplex RNA with a *k*_unw_^max^ = 0.195 ± 0.014 min^-1^ (Fig 4), making it a comparable, albeit somewhat slower, helicase than Mtr4, which displayed *k*_unw_^max^ = 0.59 ± 0.05 min^-1^ using the same substrate [23]. Additionally, FRH displays a higher rate of unwinding and functional affinity for the poly(A) substrate over a non(A) substrate (*K*_1/2 poly(A)_ = 140 ± 40 nM, *K*_1/2 non(A)_ = 615 ± 206 nM) (Fig 4), similar to that observed in Mtr4 [29]. Thus, despite the acquisition of circadian clock associated functionality, FRH retains the characteristic unwinding activity associated with Mtr4, including a discernable sequence preference for a 3’ poly(A) substrate.

The new FRH^Trig^ structure provides an example of how protein-protein interactions (in this case, a crystal contact) can dramatically alter the arch conformation. The emerging view is that protein-protein interactions between the arch domain and accessory proteins such as Nop53, Utp18 or Air2 often play an important role in recruiting Mtr4 (and presumably FRH) to RNA substrates [22, 25, 30]. It seems likely that protein and/or RNA interactions with the arch affect arch conformation, although it is unclear what impact arch dynamics may have on helicase function. In the case of FRH, interactions between the arch and the WCC may similarly affect arch conformation, which could influence the interaction with other clock proteins such as FRQ, CK1a and CKII.

## Materials and methods

### Protein expression and purification

Full length FRH DNA sequence from *N*. *crassa* was inserted into a pDB.GST vector (DNASU) using NdeI and EcoRI sites to create a GST-FRH fusion protein with a cleavable TEV target sequence linker. GST-FRH protein was recombinantly expressed in an *E*. *coli* BL21(DE3)-RIPL cell line (Stratagene). Cell lysis was performed by lysozyme treatment and sonication of cell pellets resuspended in a 20 mM sodium phosphate pH 7.5, 50 mM NaCl, 5% glycerol, and 5 mM β-mercaptoethanol (BME) lysis buffer supplemented with lysozyme, pepstatin, leupeptin, aprotinin, and PMSF. After cell lysis, the soluble fraction was incubated with Glutathione agarose resin (GoldBio) for 2 hours, followed by washing with lysis buffer and elution in 50 mM Hepes pH 7.5, 50 mM NaCl, 5% glycerol, 2 mM BME, and 20 mM reduced L-glutathione. The protein was further purified using a Heparin affinity column (GE), followed by overnight cleavage of the GST tag using TEV protease. The cleaved FRH was then purified over a DEAE column (GE). Finally, FRH was separated from GST and TEV using a Superdex 200 26/60 size exclusion column (GE) in a buffer containing 50 mM Hepes pH 7.5, 100 mM NaCl, 5% glycerol, and 2 mM BME. After sizing, FRH fractions were assessed for RNase contamination using the RNaseAlert® kit (IDT). Pure, RNase-free FRH protein was then concentrated and immediately used for crystallization trials or flash frozen in small aliquots for further analysis.

### RNA substrates

RNAs used in this study were purchased from Integrated DNA Technologies (IDT). The substrate sequences are as follows with duplex regions underlined: R16 (top strand of both substrates), 5′-AGCACCGUAAAGACGC-3′; poly(A) overhang, 5′-GCGUCUUUACGGUGCUUAAAAA-3′; non(A) overhang, 5′-GCGUCUUUACGGUGCUUGCCUG-3′. The 16 nucleotide top strand was radiolabeled using γ-^32^P ATP and T4 polynucleotide kinase and quenched by heating to 95°C before annealing, as described previously [23, 29]. The RNA substrates were purified by native polyacrylamide gel electrophoresis, gel extraction and ethanol precipitation.

### Unwinding assay

Pre-steady state unwinding activity was measured as previously described for Mtr4 [23, 29]. Briefly, various concentrations of FRH were incubated with ~0.2 nM radiolabeled RNA at 30°C in a heating block in a buffer containing 40 mM MOPS (pH 6.5), 100 mM NaCl, 0.5 mM MgCl_2_, 5% glycerol, 0.01% nonidet-P40 substitute, 2 mM DTT and 1 U/μl RiboLock RNase Inhibitor (Thermo Fisher). Reactions were initiated by the addition of equimolar ATP and MgCl_2_ at saturating concentrations. Aliquots were then removed and quenched at the indicated times at a 1:1 ratio with buffer containing 1% SDS, 5 mM EDTA, 20% glycerol, 0.1% bromophenol blue and 0.1% xylene cyanol. Samples were applied to a native 15% acrylamide TTE gel and duplex and single-stranded RNAs were separated at 120 V for 75 min. Gels were wrapped in cellophane and exposed to a phosphor screen at -80°C overnight before visualizing using a Storm Phosphorimager (Amersham Biosciences). Bands were quantified using ImageQuant software and unwinding rate constants were calculated as described [31].

Data were collected in triplicate and fit as previously described (Fraction unwound = *A*(1-exp(-*k*_unw_*t))) [23, 29]. The *k*_unw_^max^ and *K*_1/2_ values were calculated using best fit curves to *k*_unw_ = *k*_unw_^max^, _E_ [E]/([E] + *K*_1/2_, _E_); where [E] is FRH concentration, *K*_1/2_, _E_ is functional affinity, and *k*_unw_^max^, _E_ is the unwinding rate constant at enzyme saturation.

### Crystallization and X-ray structure solution

FRH was crystallized by hanging drop vapor diffusion in 0.2 M Sodium Citrate pH 5.6, 19% PEG 3350 at room temperature, with 10.5 mg/ml FRH (1:2 protein:well drop ratio). Crystals were transferred to a stabilization solution containing the well solution and 15% glycerol, then flash frozen in liquid Nitrogen.

Crystallographic data for full-length FRH crystals were collected to 3.5 Å on beamline 14–1 at the Stanford Synchrotron Radiation Lightsource (SSRL, Table 1). Data were processed using HKL2000 [32]. The crystal belongs to space group P 3_1_21 and contains one molecule in the asymmetric unit (Mathews coefficient– 2.9 Å^3^/Da, 58%). The FRH structure was solved by molecular replacement using Mtr4 as a search model (PDB ID 4U4C). The initial maps revealed a significant repositioning of the arch domain. The domain was rebuilt and modifications to the rest of the structure were completed through iterative rounds of model building and refinement. Final refinement involved positional, individual b-factor, and TLS refinement utilizing secondary structure restraints and reference model restraints using Mtr4 (PDB ID 4U4C) as a reference model [22]. Phaser-MR [33] and Phenix.refine [34] as implemented in the PHENIX software package [35] was used for molecular replacement and refinement, respectively. Model building was performed using Coot [36]. Structure validation was performed using Molprobity [37]. Final refinement statistics are shown in Table 1.

### Analytical ultracentrifugation

Sedimentation velocity analytical ultracentrifugation (SV-AUC) experiments were performed using an Optima XL-I (Beckman Coulter) analytical ultracentrifuge. Purified full length FRH was analyzed at 2.9 mg/ml, 1.5 mg/ml, 0.5 mg/ml and 0.25 mg/ml. Protein solution and a reference buffer were loaded into Beckman charcoal-epon two sector cells with 12 mm path lengths and quartz windows. The samples were analyzed at 42,000 RPM and 20°C using absorbance at 280 nm until complete sedimentation was achieved. The data were regularized with a confidence interval of 0.95 and analyzed using Sedfit [38] with a continuous c(s) distribution and 72 scans. The FRH partial specific volume, buffer density, and viscosity used in the analysis (0.73872 ml/g, 1.020700 g/ml, and 0.012083 Poise, respectively) were calculated using Sednterp [39]. The final reported values are the average and standard deviation calculated from three runs at different concentrations, further validating that no changes in oligomeric state occurs as concentrations increase.

## Supporting information

S1 Fig Updated sequence and secondary structure alignment of *nc*FRH and *sc*Mtr4. Previous *N*. *crassa* FRH and *S*. *cerevisiae* Mtr4 sequence alignments [19, 20] have been modified based on careful analysis of the existing FRH and Mtr4 structures. Observed secondary structure is displayed above and below the corresponding sequences. Regions lacking structural information are shown as dashed lines. Helix and strand numbering is included to aid future referencing of structural features. Structures used in optimization of sequence alignment include: FRH (PDB ID: 4XGT and 6BB8) and Mtr4 (PDB ID: 4QU4 and 4U4C).
(PDF)
