# Supplementary material for: Structure of frequency-interacting RNA helicase from Neurospora crassa reveals high flexibility in a domain critical for circadian rhythm and RNA surveillance
Source: PLoS One. 2018 May 2;13(5):e0196642. doi: 10.1371/journal.pone.0196642 (PMC5931499; doi:10.1371/journal.pone.0196642)
Supplement: S1 Fig — Previous N. crassa FRH and S. cerevisiae Mtr4 sequence alignments [19, 20] have been modified based on careful analysis of the existing FRH and Mtr4 structures. Observed secondary structure is displayed above and below the corresponding sequences. Regions lacking structural information are shown as dashed lines. Helix and strand numbering is included to aid future referencing of structural features. Structures used in optimization of sequence alignment include: FRH (PDB ID: 4XGT and 6BB8) and Mtr4 (PDB ID: 4QU4 and 4U4C). (PDF) [file pone.0196642.s001.pdf]

S1 Figure. Structural Alignment

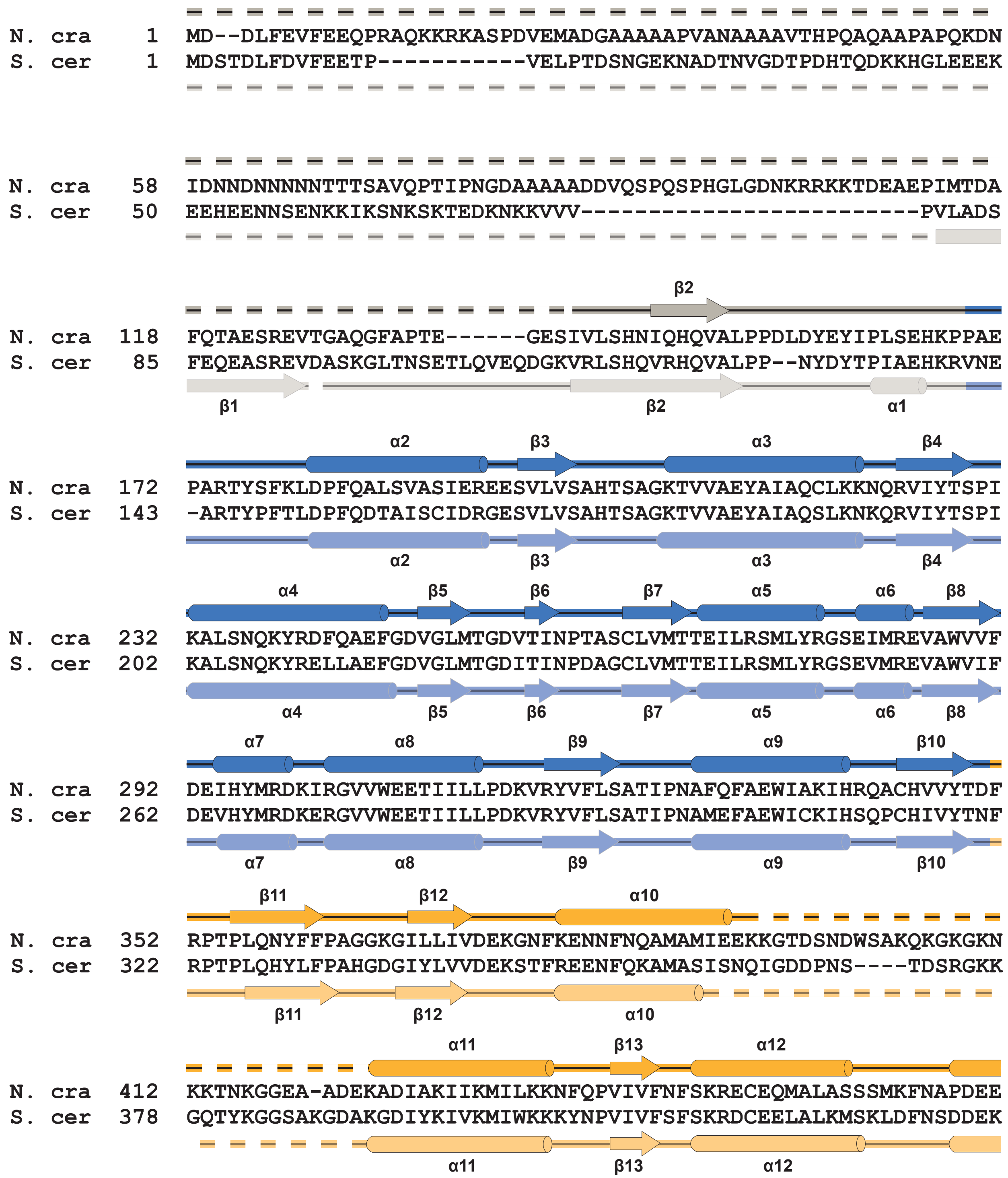

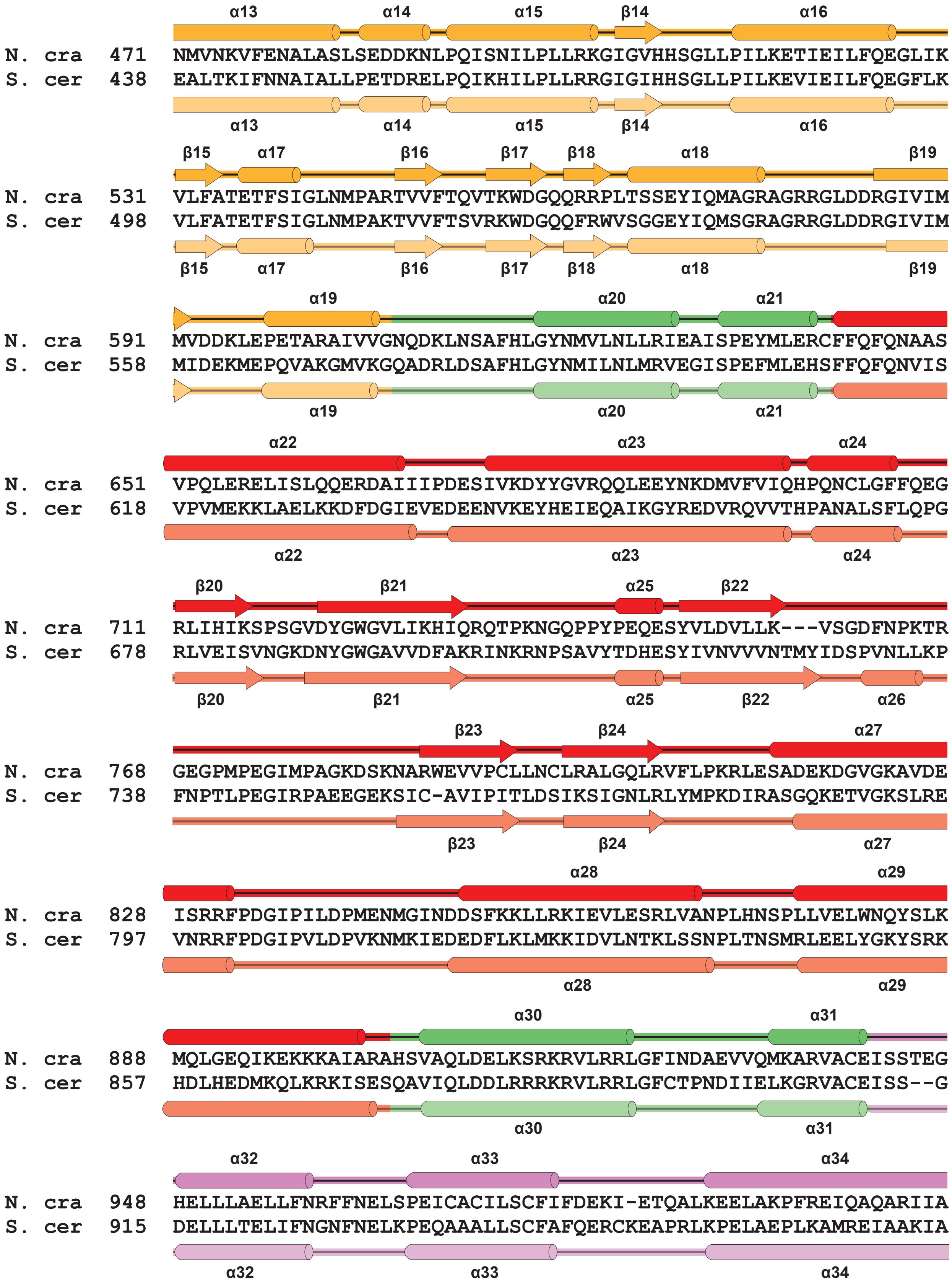
α13 α14 α15 β14 α16
N. cra 471 NMVNKVFENALASLSEDDKNLPQISNILPLLRKGIGVHHSGLLPILKETIEILFQEGLIK
S. cer 438 EALTKIFNNAIALLPETDRELPQIKHILPLLRRGIGIHHSGLLPILKEVIEILFQEGFLK
α13 α14 α15 β14 α16
β15 α17 β16 β17 β18 α18 β19
N. cra 531 VLFATETFSIGLNMPARTVVFTQVTKWDGQQRRPLTSSEYIQMAGRAGRRGLDDRGIVIM
S. cer 498 VLFATETFSIGLNMPAKTVVFTSVRKWDGQQFRWVSGGEYIQMSGRAGRRGLDDRGIVIM
β15 α17 β16 β17 β18 α18 β19
α19 α20 α21
N. cra 591 MVDDKLEPETARAIVVGNQDKLNSAFHLGYNMVLNLLRIEAISPEYMLERCFFQFQNAAS
S. cer 558 MIDEKMEPQVAKGMVKGQADRLDSAFHLGYNMILNLMRVEGISPEFMLEHSFFQFQNVIS
α19 α20 α21
α22 α23 α24
N. cra 651 VPQLERELISLQQERDAIIIPDESIVKDYYGVRQQLEEYNKDMVFVIQHPQNCLGFFQEG
S. cer 618 VPVMEKKLAELKKDFDGIEVEDEENVKEYHEIEQAIKGYREDVRQVVTHPANALSFLQPG
α22 α23 α24
β20 β21 α25 β22
N. cra 711 RLIHIKSPSGVDYGWGVLIKHIQRQTPKNGQPPYPEQESYVLDVLLK---VSGDFNPKTR
S. cer 678 RLVEISVNGKDNYGWGAVVDFAKRINKRNPSAVYTDHESYIVNVVVNTMYIDSPVNLLKP
β20 β21 α25 β22 α26
β23 β24 α27
N. cra 768 GEGPMPEGIMPAGKDSKNARWEVVPCLLNCLRALGQLRVFLPKRLESADEKDGVGKAVDE
S. cer 738 FNPTLPEGIRPAEEGEKSIC-AVIPITLDSIKSIGNLRLYMPKDIRASGQKETVGKSLRE
β23 β24 α27
α28 α29
N. cra 828 ISRRFPDGIPILDPMENMGINDDSFKKLLRKIEVLESRLVANPLHNSPLLVELWNQYSLK
S. cer 797 VNRRFPDGIPVLDPVKNMKIEDEDFLKLMKKIDVLNTKLSSNPLTNSMRLEELYGKYSRK
α28 α29
α30 α31
N. cra 888 MQLGEQIKEKKKAIARAHSVAQLDELKSRKRVLRRLGFINDAEVVQMKARVACEISSTEG
S. cer 857 HDLHEDMKQLKRKISESQAVIQLDDLRRRKRVLRRLGFCTPNDIIELKGRVACEISS--G
α30 α31
α32 α33 α34
N. cra 948 HELLLAELLFNRFFNELSPEICACILSCFIFDEKI-ETQALKEELAKPFREIQAQARIIA
S. cer 915 DELLLTELIFNGNFNELKPEQAAALLSCFAFQERCKEAPRLKPELAEPLKAMREIAAKIA
α32 α33 α34

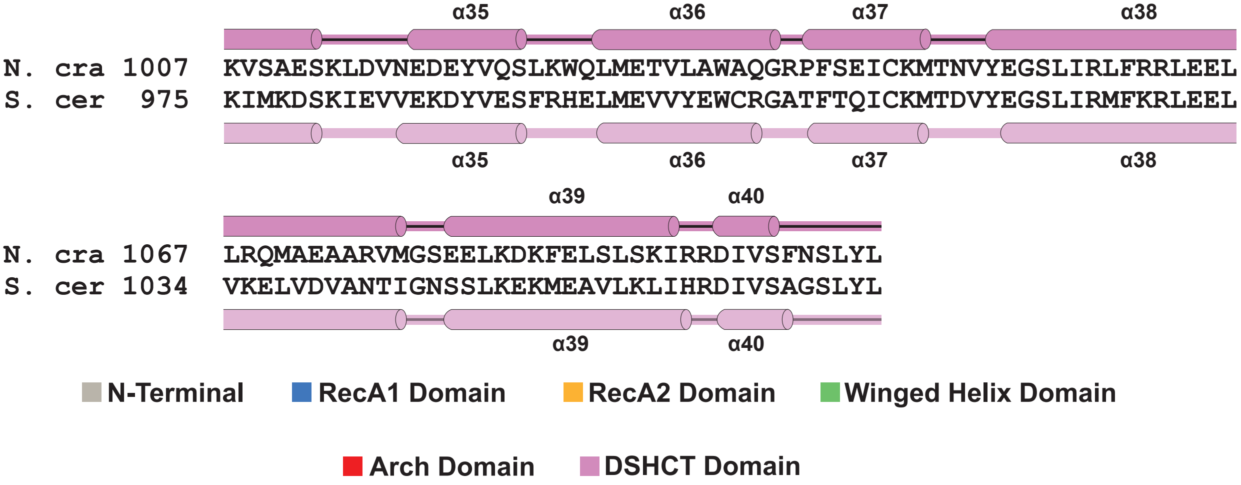

S1 Figure. Updated Sequence and Secondary Structure Alignment of ncFRH and scMtr4. Previous N. crassa-FRH and S. cerevisiae Mtr4 sequence alignments [19, 20] have been modified based on careful analysis of the existing FRH and Mtr4 structures. Observed secondary structure is displayed above and below the corresponding sequences. Regions lacking structural information are shown as dashed lines. Helix and strand numbering is included to aid future referencing of structural features. Structures used in optimization of sequence alignment include: FRH (PDB ID: 4XGT and 6BB8) and Mtr4 (PDB ID: 4QU4 and 4U4C).
